# Mining of Oil Content Genes in Recombinant Maize Inbred Lines with Introgression from Temperate and Tropical Germplasm

**DOI:** 10.3390/ijms251910813

**Published:** 2024-10-08

**Authors:** Mengfei Shi, Jiachen Sun, Fuyan Jiang, Ranjan K. Shaw, Babar Ijaz, Xingming Fan

**Affiliations:** 1 Institute of Food Crops, Yunnan Academy of Agricultural Sciences, Kunming 650205, China; smf5040@163.com (M.S.); jiachens5046@163.com (J.S.); jiangfuyansxx@126.com (F.J.); ranjanshaw@gmail.com (R.K.S.); babarijazpbg@gmail.com (B.I.); 2 College of Agronomy and Biotechnology, Yunnan Agricultural University, Kunming 650201, China

**Keywords:** maize, total oil content, quantitative trait locus mapping, genome-wide association study, candidate genes

## Abstract

The oil content of maize kernels is essential to determine its nutritional and economic value. A multiparent population (MPP) consisting of five recombinant inbred line (RIL) subpopulations was developed to elucidate the genetic basis of the total oil content (TOC) in maize. The MPP used the subtropical maize inbred lines CML312 and CML384, along with the tropical maize inbred lines CML395, YML46, and YML32 as the female parents, and Ye107 as the male parent. A genome-wide association study (GWAS) was performed using 429 RILs of the multiparent population across three environments, employing 584,847 high-quality single nucleotide polymorphisms (SNPs). Furthermore, linkage analysis was performed in the five subpopulations to identify quantitative trait loci (QTL) linked to TOC in maize. Through QTL mapping and GWAS, 18 QTLs and 60 SNPs that were significantly associated with TOC were identified. Two novel candidate genes, *Zm00001d029550* and *Zm00001d029551*, related to TOC in maize and located on chromosome 1 were reported, which have not been previously reported. These genes are involved in biosynthesis, lipid signal transduction, plant development and metabolism, and stress responses, potentially influencing maize TOC. Haplotype analysis of *Zm00001d029550* and *Zm00001d029551* revealed that Hap3 could be considered a superior haplotype for increasing TOC in maize. A co-located SNP (SNP-75791466) on chromosome 1, located 5648 bp and 11,951 bp downstream of the candidate genes *Zm00001d029550* and *Zm00001d029551*, respectively, was found to be expressed in various maize tissues. The highest expression was observed in embryos after pollination, indicating that embryos are the main tissue for oil accumulation in maize. This study provides a theoretical basis for understanding the genetic mechanisms underlying maize TOC and developing high-quality, high-oil maize varieties.

## 1. Introduction

Maize (*Zea mays* L.) is an important crop grown across various regions worldwide and serves as a significant source of vegetable oil. Due to its relatively large seeds and embryos, as well as its high unsaturated fatty acid content, maize produces high-quality oil [1,2,3]. Among the three main components of maize kernels, oil provides 2.25 times more energy than starch [4]. Maize oil primarily consists of five fatty acids, which constitute over 98% of its total oil content: palmitic (C16:0), stearic (C18:0), oleic (C18:1), linoleic (C18:2), and linolenic (C18:3) acids [5]. However, compared to other oilseed crops, maize seeds possess relatively low oil content. Therefore, plant breeders aim to increase the oil percentage by analyzing different maize genotypes and their oil accumulation. Noel et al. [6] reported significant variations in protein, starch, and oil content among tropical maize populations, with oil content ranging from 4.5% to 6.2%. Hybrids with high oil content in maize (>6%) are considered valuable due to their nutrient content [7]. Various genetic resources have been developed through extensive artificial selection of high-oil maize populations [8]. Beijing High Oil (BHO), a high-oil maize population, originated from the synthetic variety Zhongzong No. 2, which was created from 12 inbred lines of Lancast heterotic groups. After 18 selection cycles, the oil content increased from 4.71% to 15.55% [9]. The oil content of the original open-pollinated variety Illinois High Oil (IHO) increased to approximately 20% following 100 generations of selection [10]. Thus, increasing the oil content remains a key objective in plant breeding and biotechnological enhancement of maize [5].

Understanding the genetic basis of oil synthesis and accumulation is crucial for providing key insights into marker-assisted selection and genetic modification to enhance the TOC. Previous studies have extensively studied the QTLs related to TOC in maize kernels [11,12,13]. Alrefai et al. [14] identified multiple QTLs that influence TOC, laying the foundation for subsequent genetic exploration. Yang et al. [11] identified several major QTLs with significant additive effects that are crucial for determining the fatty acid composition and increasing oil content in the studied germplasm. Additionally, numerous minor QTLs and some epistatic QTLs, all with additive effects, also influenced the fatty acid composition and oil content. Alleles from the high-oil parent “By804” had a positive impact on TOC across all mapped loci, indicating that the oil content can be enhanced through the selection of beneficial genes. Zhang et al. [15] identified 16 QTLs associated with TOC in maize. The moderate to high broad-sense heritability (67.00–86.60%) indicated that genetic factors largely influence the variations in TOC. Overall, previous findings indicate that TOC, as a quantitative trait, is influenced by gene–environment interactions. Therefore, identifying QTLs related to maize grain traits to uncover the causal variations regulating oil content will offer valuable insights into characterizing the genes associated with kernel oil content. This can be achieved through map-based cloning or candidate gene association mapping. GWAS has been successfully employed to identify SNPs and candidate genes related to maize grain-related traits, offering valuable insights into maize’s genetic structure [16,17,18,19]. For instance, Belo et al. [20] used GWAS to identify a candidate locus on chromosome 4 that significantly affected TOC in maize. Li et al. [5] conducted GWAS using 368 maize inbred lines with 1.03 million SNPs, revealing 74 loci significantly associated with TOC. In summary, numerous studies have successfully identified SNPs associated with TOC, underscoring the utility and efficiency of GWAS [21,22,23].

Developing subtropical and tropical germplasm resources is crucial for breeding core germplasms and addressing homogenization issues in the seed industry. Tropical and subtropical maize germplasms generally have higher oil content compared to temperate germplasms. The increased oil content helps them adapt to warmer climates and contributes to their nutritional profiles. In this study, five subtropical and tropical elite inbred lines (CML312, CML384, CML395, YML46, and YML32) with broad genetic variation and high oil content were selected as donor parents from the Reid, Non-Reid, and Suwan heterotic groups. These inbred lines were crossed with the elite inbred line Ye107 from the Reid heterotic group to develop a multiparent population (MPP) comprising 429 RILs. The International Maize and Wheat Improvement Center (CIMMYT) tropical maize germplasm possesses the richest genetic diversity globally. One of the female parents used in this study, the tropical germplasm CML395, was bred by CIMMYT and is extensively used in maize breeding due to its excellent combining ability. Against this backdrop, the present study was conducted with the following objectives: (1) to identify significant QTLs and SNPs associated with maize TOC across five RIL subpopulations in different environments, and (2) to further identify novel candidate genes regulating maize TOC in tropical germplasms.

## 2. Results

### 2.1. Phenotypic Analysis of TOC in Five RIL Subpopulations

The phenotypic analysis of the five subpopulations of the MPP for TOC was performed in three different environments: 22YS, 23JH, and 21YS, and the relevant data were collected. The descriptive statistics of TOC for the five RIL subpopulations are presented in Table 1, and the coefficient of variation (CV) for each subpopulation across the three environments was calculated. The skewness and kurtosis for all five RIL subpopulations were below 1, indicating minimal bias. The frequency distribution of TOC in each of the five subpopulations followed a near-normal distribution. Under three different environmental conditions, broad-sense heritability for TOC was high in pop2 (93.1%) and pop5 (91.6%). Additionally, the genotype × environment interaction variances were statistically significant.

**Figure 1:**
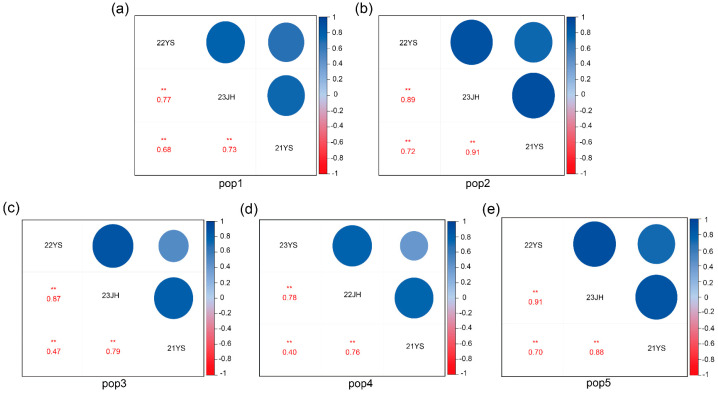
Correlations between pop1, pop2, pop3, pop4, and pop5 for TOC in three environments. (**a**) Correlation of pop1 for TOC between the 22YS, 23JH, and 21YS environments; (**b**) correlation of pop2 for TOC between the 22YS, 23JH, and 21YS environments; (**c**) correlation of pop3 for TOC between the 22YS, 23JH, and 21YS environments; (**d**) correlation of pop4 for TOC response between the 22YS, 23JH, and 21YS environments; (**e**) correlation of pop5 for TOC between the 22YS, 23JH, and 21YS environments. ** indicates *p* < 0.01.

The correlation analysis of TOC for the RIL subpopulations across different environments is presented in Table 1. For pop1, the correlation coefficient between the 22YS and 23JH environments for TOC was 0.77, between the 23JH and 21YS was 0.73, and between the 21YS and 22YS environments was 0.68 (Figure 1a). For pop2, the correlation coefficient between the 22YS and 23JH environments was 0.89, between the 23JH and 21YS environments was 0.91, and between the 21YS and 22YS environments was 0.72 (Figure 1b). For pop3, the correlation coefficient between the 22YS and 23JH environments for TOC was 0.87, between the 23JH and 21YS environments was 0.79, and between the 21YS and 22YS environments was 0.47 (Figure 1c). For pop4, the correlation coefficient between the 22YS and 23JH environments was 0.78, between the 23JH and 21YS environments was 0.76, and between the 21YS and 22YS environments was 0.40 (Figure 1d). For pop5, the correlation coefficient between 22YS and 23JH was 0.91, between 23JH and 21YS environments was 0.88, and between the 21YS and 22YS environments was 0.70 (Figure 1e). The consistently high correlation coefficients among the three environments suggested that the TOC of the RILs of the five subpopulations were stable across different environmental conditions, thereby ensuring the reliability of the subsequent GWAS analysis.

### 2.2. Phylogenetic Tree, PCA, and Population Structure Analysis

**Figure 2:**
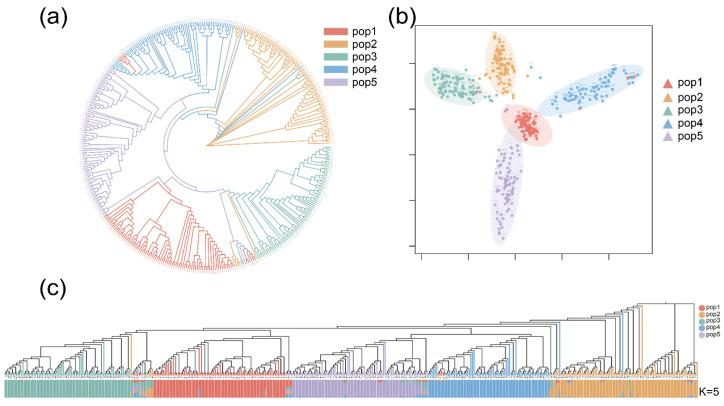
Genetic diversity analysis of the 429 RILs of the MPP. (**a**) Phylogenetic tree. (**b**) Principal component analysis. (**c**) Bayesian clustering plots of 429 maize RILs at K = 5.

The principal component analysis (PCA) results showed that the 429 RILs were grouped into five clusters, which was consistent with the experimental design of this study (Figure 2b). The scattered points in the PCA plot may have arisen due to within-population heterogeneity or outliers. Phylogenetic analysis revealed that the 429 RILs were predominantly clustered into five subgroups, which corresponded to the PCA results (Figure 2a). Population structure analysis conducted using the Admixture software Version 1.3 [24] showed that the 429 RILs were categorized into five subpopulations at K = 5 (Figure 2c). The admixture between the subgroups may have resulted from genetic drift or natural hybridization. Overall, the results of the phylogenetic analysis, PCA, and population structure were consistent.

### 2.3. LD Decay Analysis

**Figure 3:**
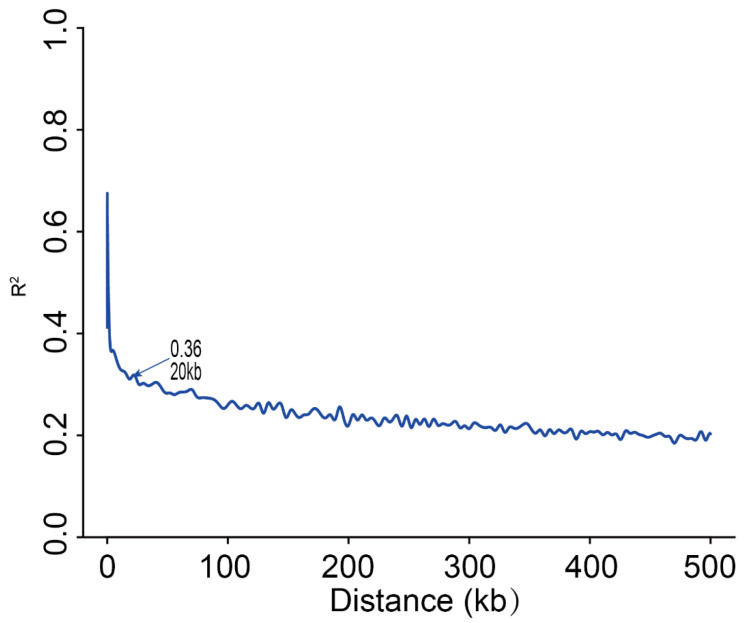
The LD decay plot of the MPP.

Genome-wide SNPs were used to assess the extent of linkage disequilibrium (LD) in the MPP, and the LD decay was measured. The LD decay was calculated for the MPP, and it was found that when r^2^ decayed by half, the physical distance was approximately 20 kb (Figure 3). The rapid LD decay indicated that the higher degree of domestication corresponded to greater selection intensity, resulting in a decrease in genetic diversity.

### 2.4. Genome-Wide Association Analysis for TOC in Maize

**Figure 4:**
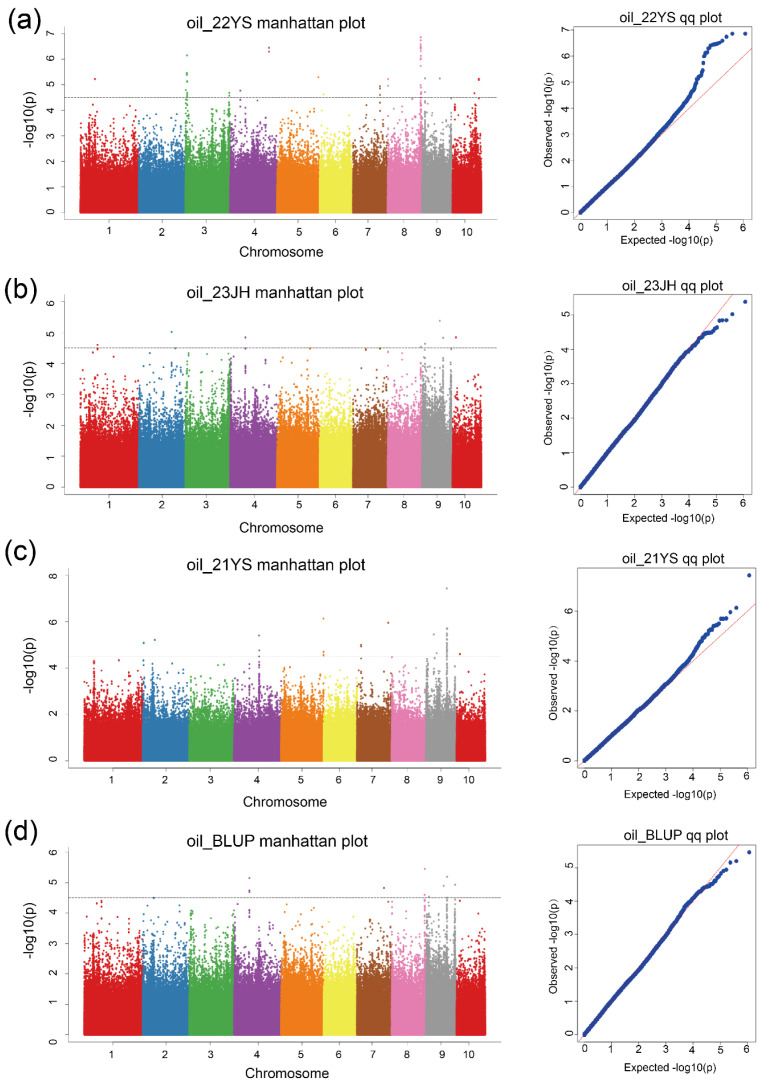
Manhattan map (**left**) and Q–Q plots (**right**) for (**a**) 22YS environment, (**b**) 23JH environ–ment, and (**c**) 21YS environment; (**d**) BLUP values indicate the SNPs associated with TOC. In the Manhattan plot, each dot represents an SNP, and the black line denotes the threshold value. Differ–ent colors in the Manhattan plot represent different chromosomes. In the Q–Q plot, the red line denotes the expected significance value, while the blue dots represent the observed significance val–ues.

GWAS was conducted using 584,847 high-quality SNPs combined with the mean TOC values of 429 RILs of the MPP across three environments. SNPs with a minimum allele frequency (MAF) ≥5% and a missing value of r^2^ < 0.8 were used for GWAS analysis. Additionally, GWAS was performed using the BLUP values of the TOC of the RILs of the MPP. SNPs exceeding a threshold value of −log10(P) > 4.5 were considered significant. The association analysis used a mixed linear model (MLM) to identify loci associated with TOC in maize. During the GWAS, a total of 60 SNPs significantly associated with TOC were identified (Table 2). In the 22YS environment, 23 significant SNPs were identified on chromosomes 1, 3, 4, 5, 6, 7, 8, 9, and 10 (Table 2, Figure 4a). The phenotypic variation explained by these SNPs ranged from 2.8–11.7%. In the 23JH environment, eight SNPs were significantly associated with TOC, distributed across chromosomes 1, 2, 4, 8, 9, and 10 (Table 2, Figure 4b). The phenotypic variation explained by these SNPs ranged from 3.4% to 8.3%. In the 21YS environment, 20 significant SNPs were identified on chromosomes 2, 4, 5, 7, 9, and 10 (Table 2, Figure 4c) accounting for 4.4–13.2% of the phenotypic variation. In the BLUP environment, nine significant SNPs were identified on chromosomes 4, 7, 8, and 9 (Table 2, Figure 4d), accounting for 3.8% to 9.4% of the phenotypic variation. Among these, four SNPs were consistently identified and found to be co-located across different environments, and seven SNPs were found to be co-located with the BLUP values. The QQ plots showed that false positives were controlled during the GWAS across all environments (Figure 4a–d).

### 2.5. Genetic Map Construction and QTL Mapping of TOC in the Five RIL Subpopulations

In the present study, high-density linkage maps were constructed for five RIL subpopulations to identify QTLs linked to TOC in maize. The genetic map of pop1 was developed using 981 polymorphic SNPs, spanning a total genetic distance of 1045.83 cM. The average genetic distance between the markers was 1.07 cM. The genetic map of pop2 was constructed using 693 polymorphic SNPs, spanning a total genetic distance of 575.78 cM, with an average genetic distance of 0.83 cM. The genetic map of pop3 was constructed using 2021 polymorphic SNPs, spanning a total genetic distance of 4953.45 cM. The average genetic distance between the markers was 2.27 cM. The genetic map of pop4 was constructed using 857 polymorphic SNPs, spanning a total genetic distance of 802.12 cM, with an average genetic distance of 0.94 cM. The genetic map of pop5 was constructed using 638 polymorphic SNPs spanning a total genetic distance of 581.28 cM. The average genetic distance between the markers was 0.91 cM.

**Figure 5:**
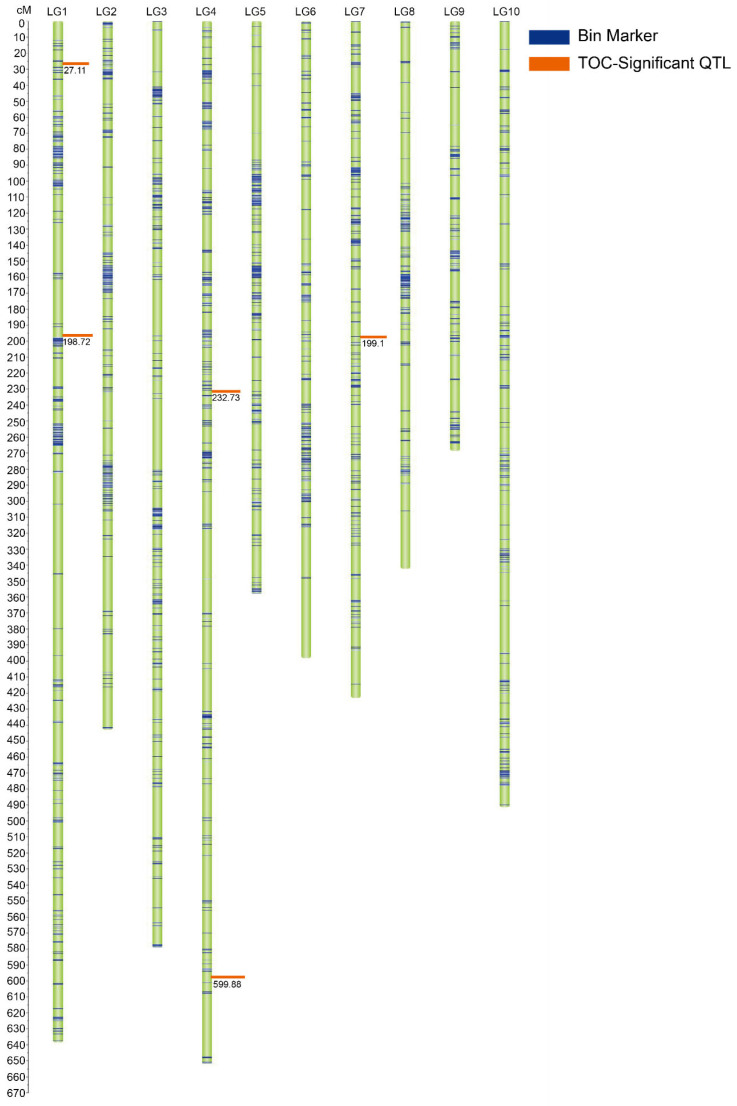
QTL mapping for TOC in Pop3. Blue bands represent the bin markers and orange boxes indicate the significant QTLs linked to TOC.

QTL mapping and effect analysis of TOC for pop1, pop2, pop3, pop4, and pop5 were conducted across the three environments. During QTL mapping, QTLs for TOC were identified with an LOD threshold of 2.5. In pop1, five QTLs (*qTOC2-1*, *qTOC2-2*, *qTOC2-3*, *qTOC3-1*, and *qTOC9-*1) linked to TOC were detected in the three environments, explaining 9%, 6%, 5%, 3%, and 3% of the phenotypic variance, respectively (Table 3). The QTLs were located on chromosomes 2, 3, and 9. The LOD of *qTOC2-2* on chromosome 2 was the highest (4.63), with an additive effect of 0.13. The additive effects of *qTOC2-1* and *qTOC2-3* were positive, indicating that these two QTLs had positive effects on TOC. No significant QTLs for TOC were identified in pop2 during linkage analysis. In pop3, five QTLs (*qTOC1-1*, *qTOC1-2*, *qTOC4-1*, *qTOC4-2*, and *qTOC7-1*) were detected, explaining 13.1%, 1.4%, 19.9%, 14.3%, and 15.1% of phenotypic variance, respectively (Table 3). These QTLs were distributed on chromosomes 1, 4, and 7 (Figure 5). The LOD of *qTOC4-1* on chromosome 4 was the highest (5.65) with an additive effect of 0.65. Overall, the additive effects of all the four QTLs were positive. In pop4, three QTLs (*qTOC5-1*, *qTOC7-1*, and *qTOC8-1*) linked to TOC were identified across the three different environments, explaining 0.5%, 11%, and 12.4% of the phenotypic variance, respectively (Table 3). These QTLs were located on chromosomes 5, 7, and 8. The LOD of *qTOC8-1* identified on chromosome 8 was the highest (4.61), with an additive effect of 0.124. The additive effect of *qTOC5-1* was found to be positive. In pop5, five QTLs (*qTOC2-1*, *qTOC2-2*, *qTOC3-1*, *qTOC5-1*, and *qTOC5-2*) were identified across the three environments, explaining 14.4%, 23.1%, 17.7%, 14.6%, and 10.3% of the phenotypic variance, respectively (Table 3). These QTLs were located on chromosomes 2, 3, and 5. Among the QTLs, *qTOC2-2* on chromosome 2 exhibited the highest LOD (5.73) and the highest additive effect (0.36). The additive effects of *qTOC2-2* and *qTOC5-1* were also positive.

### 2.6. Identification of Candidate Genes Related to TOC and Haplotype Analysis

**Figure 6:**
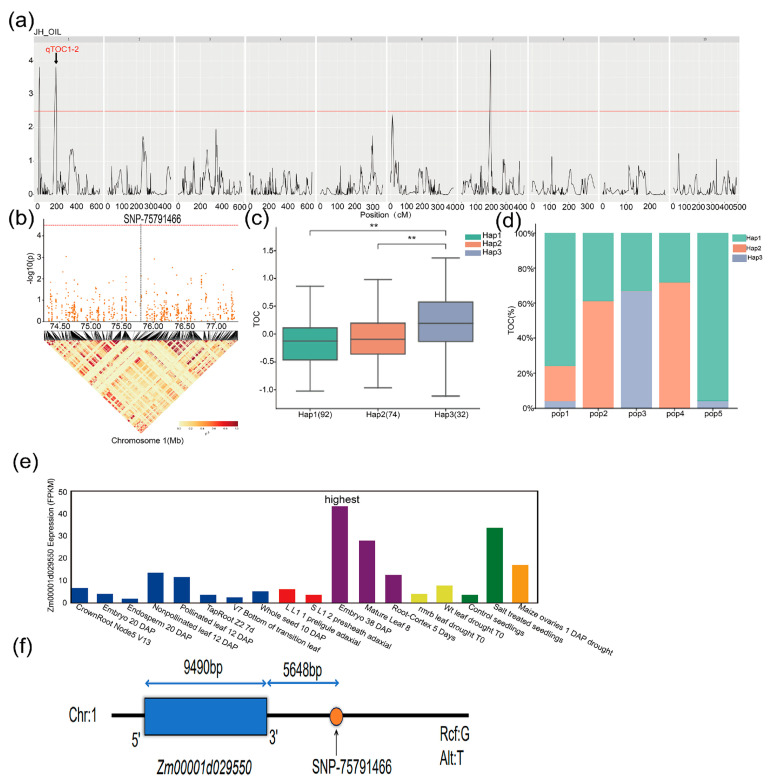
The identification of candidate genes related to maize TOC (**a**) QTLs identified on different chromosomes of maize in the 23JH environment in pop3. (**b**) The location of the most significant SNPs on chromosome 1, as identified through GWAS. (**c**) Haplotype analysis of candidate gene *Zm00001d029550* for TOC in the MPP. (**d**) The haplotype distribution of *Zm00001d029550* in five subpopulations, ** represents *p* ≤ 0.01. (**e**) The expression levels (FPKM) of the candidate gene *Zm00001d029550* in different tissues, with the highest level observed in embryos after pollination. (**f**) The position of *Zm0001d029550* and the associated SNP.

In this study, QTL mapping and GWAS analyses were used to identify the loci associated with the TOC of maize. Both analyses were compared to reveal the candidate genes that regulate TOC in maize. One significant QTL, *qTOC1-2*, detected on chromosome 1 in pop3 in the 23JH environment (Figure 6a) explained 1.4% of the phenotypic variation for TOC. The SNP-75791466 identified through GWAS in the 22YS environment overlapped the QTL interval of *qTOC1-2* (Table 4, Figure 6b) on chromosome 1. The SNP was highly significant with a −log^10^(p) value of 5.24. Based on functional annotations, two candidate genes associated with TOC, *Zm00001d029550* and *Zm00001d029551* were identified in close proximity to SNP-75791466. The SNP-75791466 was located 5648 bp downstream of *Zm00001d029550* (Figure 6f) and 11,951 bp downstream of *Zm00001d029551* (Figure 6d). *Zm00001d029550* acts as a secondary messenger of diacylglycerol (DAG) as a protein kinase C activator.

Haplotype analysis of the candidate genes was performed to identify the dominant haplotypes related to TOC. The gene *Zm00001d029550* exhibited three haplotypes: Hap1, Hap2, and Hap3 (Figure 6c, Table 5). The frequency distribution of Hap1 in the MPP was 92, Hap2 was 74, and Hap3 was 32 out of the 429 RILs. Hap3 exhibited a significantly higher TOC than Hap1 and Hap2, whereas Hap1 showed the lowest TOC in the MPP (Figure 6c). Hap3 was considered a superior haplotype of *Zm00001d029550* for increasing TOC in maize. As shown in Figure 6d, the Hap1 haplotype was present in pop1, pop2, pop3, pop4, and pop5. Hap2 was found in pop1, pop2, and pop4, whereas Hap3 was present in pop1, pop3, and pop5. The expression of *Zm00001d029550* was relatively low across various tissues, with the highest expression observed in embryos after maize pollination (Figure 6e), indicating that embryos are the primary tissue for oil accumulation in maize, which is likely to be associated with oil synthesis and metabolism.

**Figure 7:**
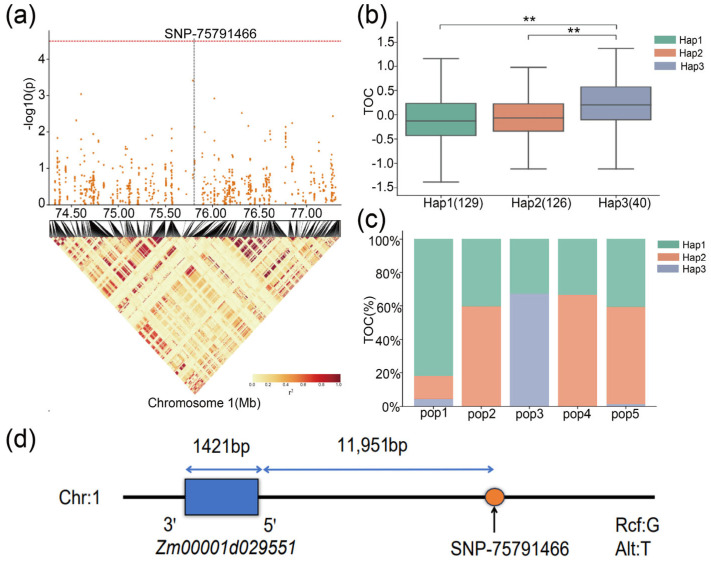
(**a**) The location of the significant SNPs on chromosome 1 identified through GWAS. (**b**) Haplotype analysis of candidate gene *Zm00001d029551* for TOC in the MPP. (**c**) The haplotype dis–tribution of *Zm00001d029551* in five subpopulations. ** represents *p* ≤ 0.01. (**d**) The position of *Zm0001d029551* and the associated SNP.

Haplotype analysis of another candidate gene, *Zm00001d029551*, was performed to identify the dominant haplotypes associated with TOC in maize. The analysis revealed that *Zm00001d029551* possessed three haplotypes: Hap1, Hap2, and Hap3 (Figure 7b, Table 5). The frequency distributions of these haplotypes among the 429 RILs were 129 for Hap1, 126 for Hap2, and 40 for Hap3. Hap3 exhibited significantly higher TOC than Hap1 and Hap2 in the MPP, whereas Hap1 exhibited the lowest TOC (Figure 7b). Therefore, Hap3 was considered the superior haplotype of *Zm00001d029551* for increasing TOC in maize. As shown in Figure 7c, the Hap1 haplotype was present in pop1, pop2, pop3, pop4, and pop5 subpopulations. Hap2 was found in pop1, pop2, pop4, and pop5, whereas Hap3 was present in pop1, pop3, and pop5.

## 3. Discussion

### 3.1. Comparison of Loci Significantly Associated with TOC with Previously Reported QTLs

In the present study, phenotypic analysis showed that TOC had a near-normal distribution across the three different environments (Table 2), suggesting that this trait may be controlled by multiple genes. Through combined QTL mapping and GWAS, a QTL, *qTOC1-2*, and a significant SNP, both associated with TOC, were identified. Notably, this QTL had previously been reported within the TOC-linked QTL interval by Yang et al. [25]. Fang et al. [8] also detected 19 QTLs related to maize TOC in an RIL population, among which one QTL related to TOC was identified on chromosome 1, corresponding to *qTOC1-2* in our study. Furthermore, the consistency of *qTOC1-2* with previous studies on TOC is evident. Yang et al. [11] identified 18 candidate genes related to maize TOC through linkage analysis in an RIL population, with a QTL linked to TOC also located on chromosome 1, overlapping the QTL interval of *qTOC1-2* identified in our study. Additionally, Zhang et al. [26] investigated the genetic regulation of the embryo-to-endosperm ratio (EER) in maize and found that *ZmGE2* was highly expressed in the embryo. This gene is located within the embryo-related QTL interval identified in our study (Table 6). The candidate genes identified in this study were located on chromosome 1 with a phenotypic variance of 1.4%. Although the chromosomal locations of these QTLs aligned with previous studies, there were differences in the intervals. Possible explanations include (1) the use of diverse germplasms across studies, which can lead to variations in identifying genomic regions controlling TOC. This study used tropical germplasms, which may introduce greater genetic diversity. (2) Experiments were conducted in both subtropical and tropical regions, where environmental conditions vary, possibly resulting in the identification of QTLs with different effects. Furthermore, (3) differences in sample size and statistical methodologies across studies can affect GWAS accuracy. This study used 429 RILs, a sample size larger than those in previous studies [27,28,29,30]. Nevertheless, the consistency of our findings with those of previous studies supports the accuracy of our results in identifying the loci associated with TOC. The SNPs identified in this study should be investigated further to provide a theoretical basis for future comprehensive research on TOC in maize.

### 3.2. Functional Annotation of Candidate Genes

Haplotype analysis was performed for the candidate genes *Zm00001d029550* and *Zm00001d029551*. Significant differences in TOC were observed among different haplotypes of *Zm00001d029550*. The analysis revealed that Hap3 was the superior haplotype associated with increased TOC and was present in subpopulations pop1, pop3, and pop5. Notably, in pop3, Hap3 accounted for over 60% of the TOC, suggesting its significant influence on oil content in this subpopulation and its potential role in regulating maize oil content. Similarly, significant differences in TOC were found among the haplotypes of *Zm00001d029551*. The superior haplotype, Hap3 was observed in pop1, pop3, and pop5, with over 60% in pop3. This further indicated that Hap3 plays a crucial role in influencing TOC in pop3 and may be important in the regulation of oil content in maize.

Candidate genes were identified by screening a 20 kb region upstream and downstream of significant SNPs using resources such as maizeGDB, InterPro, and NCBI databases, along with relevant published research. Through comprehensive screening, two candidate genes (*Zm00001d029550* and *Zm00001d029551*) were identified on chromosome 1, with one of them functionally annotated. *Zm00001d029550* encodes diacylglycerol kinase (DGK), a crucial enzyme in the lipid signaling pathway that mediates signal transmission from hormones, neurotransmitters, immunological factors, and growth factors. This gene is involved in various processes, including biosynthesis, lipid signal transduction, phosphatidylinositol synthesis, plant development and metabolism, and stress responses. Subcellular localization of DGK genes has shown their widespread distribution across various cell compartments, with movement across multiple organelle membranes. DGK genes have been reported in nearly all plant tissues at various developmental stages. In Arabidopsis, the protein-encoding domain organizations of *AtDGK1* and *AtDGK2* are similar, and both are classified under Cluster I of plant DGKs. *AtDGK1* cDNA is primarily expressed in roots, shoots, and leaves [31], whereas the *AtDGK2* transcripts are expressed throughout the plant, except in stems [32]. In the inflorescence and floral tissues, *AtDGK3* (cluster II), *AtDGK4* (cluster II), and *AtDGK5* (cluster III) are expressed in petals, stamens, and pistils, with particularly high expression observed in the stamens [33,34]. Another study on maize identified the presence of DGKs in various plant tissues throughout the reproductive, vegetative, and developmental stages. These tissues include the stems, roots, seedlings, elongation stage, huge bellbottom stage, tasseling stage, endosperm, and mature seeds [35].

Searches in MaizeGDB and NCBI did not reveal functional annotations for *Zm00001d029551*, which overlapped with *Zm00001d029550*, suggesting a potential functional similarity. Previous studies have indicated that in maize chloroplasts, two genes may overlap by several nucleotides and be transcribed divergently from complementary DNA strands [36], implying that overlapping and functionally similar genes exist. Therefore, we hypothesized that *Zm00001d029551* may share an overlapping region with *Zm00001d029550*, indicating a functional similarity between *Zm00001d029551* and *Zm00001d029550*. These findings offer a theoretical foundation for further exploration of the genetic mechanisms underlying TOC accumulation in maize. This improved our understanding of TOC regulation in maize and supported the development of maize varieties and hybrids with high oil content.

### 3.3. Mechanism of Synthesis of TOC in Maize

This study used tropical maize germplasms, which may affect the synthesis of TOC in maize, considering that tropical regions usually experience warm and humid conditions. The impact of environmental factors on the TOC in maize is a complex and important area of research. In addition to the slight increase in maize grain TOC with the application of N, P, and K fertilizers [37], other environmental factors may also affect TOC. For example, factors such as soil type and moisture conditions may influence the TOC in maize grains, possibly indirectly through their impact on plant growth and metabolic processes. Additionally, genetic factors also play a role in determining the TOC of maize kernels. The synthesis of maize oil involves multiple biochemical pathways, including those for fatty acid, diacylglycerol, and glycerol-phospholipid. These pathways are influenced by various regulatory elements, including transcription factors, hormones, and nutrients. Studies have shown that certain key genes are crucial in oil synthesis pathways, and their expression levels may be regulated by long-term artificial selection, thereby affecting both the rate and yield of oil synthesis [26].

Previous studies have shown a high broad-sense heritability for maize grain oil-related traits (52–98%) [8,11], indicating a significant effect of genetic factors on TOC. The oil content of the ultra-high-oil maize line ‘HuajianF’ exceeds that of the Illinois high-oil maize line by 5%, which was developed after 100 generations of selection [38]. This suggests that differences in genotypes can result in significant variations in the TOC of maize grains. Moreover, grain morphology may influence TOC. Studies have found that shrunken maize grains exhibit higher oil content than round maize grains, indicating a correlation between grain shrinkage and increased oil content. Since most maize grain oil is concentrated in the embryo, seed oil content is primarily determined by the oil content of the embryo and the proportion of the embryo to the seed, further explaining the influence of morphology on TOC.

Several effective strategies have been proposed to better understand and enhance the oil content of maize in tropical regions. First, appropriate management practices, such as irrigation, fertilization, and dense planting, have been shown to increase the growth rate of maize plants and improve nutrient utilization efficiency, thereby promoting oil synthesis and accumulation, effectively increasing the oil content of maize [39]. Secondly, selecting maize varieties with stronger adaptability and tolerance to high temperature and humidity under adverse environmental conditions can help maintain higher growth rates and oil synthesis efficiency, leading to increased oil content. Furthermore, applying biotechnological methods to regulate maize oil synthesis is another important approach for enhancing oil content. Increasing enzyme levels through selective breeding and/or genetic manipulation can boost fatty acid (FA) content in maize embryos, thereby enhancing oil content [40]. In summary, oil synthesis in maize in tropical regions is a complex biochemical process influenced by environmental, genetic, and regulatory factors. Further research on the interactions among these factors will help uncover the mechanisms underlying maize oil formation, providing a scientific basis and technical support for increasing maize yield and quality in tropical regions.

### 3.4. Genetic Effects of Oil Content in Tropical Maize

Studies have shown that two kernel composition studies, which utilized either RILs or S2 lines from the same source, displayed differing levels of epistasis for oil content in maize [41,42]. Laurie et al. used RILs and found that variation in oil content was largely attributed to additive effects, leaving minimal scope for detecting epistatic effects [41]. In contrast, a study conducted by Dudley used S2 progenies and observed a greater degree of oil variation due to dominant genetic effects and identified significant nonadditive epistatic interactions [42]. In summary, additive, dominant, and overdominance effects collectively influence oil content in tropical maize. Therefore, a deeper understanding of these genetic effects is crucial when applying genetic improvement methods to enhance oil content in tropical maize. Future research should focus on exploring gene interactions and their specific roles in the genetic regulatory network of tropical maize, aiming for more efficient utilization of genetic resources to increase maize yield and quality.

This study offers significant insights into the genetic and environmental factors influencing total oil content (TOC) in maize. Our analysis, combining QTL mapping and GWAS, identified an important QTL, *qTOC1-2*, and a key SNP associated with TOC on chromosome 1, corroborating the findings from previous studies. This QTL overlaps with an interval of QTLs identified in earlier studies, underscoring the role of genetic diversity and environmental conditions in TOC variation. The identified candidate gene, *Zm00001d029550*, which encodes diacylglycerol kinase (DGK), further elucidates the biochemical pathways involved in oil synthesis. Haplotype analysis suggested that specific variants, such as Hap3, are crucial for enhancing TOC. Additionally, investigation into environmental and genetic effects highlights the complex interplay influencing maize oil content. These findings provide a strong foundation for future research aimed at optimizing maize oil content, particularly in tropical regions, through improved genetic strategies and management practices.

## 4. Materials and Methods

### 4.1. Plant Materials and Population Development

In this study, the subtropical maize inbred lines CML312 and CML384, along with the tropical maize inbred lines CML395, YML46, and YML32, were used as female parents, whereas the temperate inbred line Ye107 served as the common male parent for hybridization. The F_1_s were self-pollinated for nine generations using the single seed descent method, and a multiparent population (MPP) comprising five RIL subpopulations was developed: pop1 (CML312 × Ye107), pop2 (CML384 × Ye107), pop3 (CML395 × Ye107), pop4 (YML46 × Ye107), and pop5 (YML32 × Ye107), resulting in a total of 429 RILs with wide genetic variation. Among the 429 RILs, pop1 and pop2 each contained 92 RILs, pop3 contained 83, pop4 contained 74, and pop5 contained 88 RILs. The pedigrees, ecotypes, and total oil content of the six parental lines are listed in Table 7. The TOC of the parental lines ranged from 3.51% (Ye107) to 7.3% (YML32) (Table 7). The common parent Ye107 is an elite inbred line derived from lines belonging to two heterotic groups used in the Chinese breeding program and has served as a parent in numerous commercial hybrids in major production areas in China. The experimental trials were conducted in three ecological environments: Yanshan (YS) County (altitude: 1540 m, longitude: 104.5° E, latitude: 23.6° N) in 2021 (21YS) and 2022 (22YS), and in Jinghong (JH) City (altitude: 606.5 m, longitude: 100.58° E, latitude: 21.54° N) in 2023 (23JH) in Yunnan Province, China.

### 4.2. Experimental Design and Oil Content Estimation

The experimental trials were conducted using a randomized complete block design (RCBD) in the 21YS, 22YS, and 23JH environments, with three replicates at each location. Each experimental plot was 4.0 m in length with a row spacing of 0.70 m and an inter-plant spacing of 25 cm, and contained 14 plants per row. Standard agronomic practices were followed during the trials.

The oil content of the maize was determined using a near-infrared grain analyzer with five replicates for each recombinant inbred line [43]. This method involves exposing seeds to infrared rays, which exhibit different levels of absorption depending on the oil content of the seeds. Standard samples of seeds with varying oil content gradients were used to obtain a standard curve, which was then applied to determine the absorption of near-infrared rays by the seeds being measured. The oil content was measured by comparing the absorption of the seeds to the standard curve.

### 4.3. Statistical Analysis of TOC and Estimation of Heritability

After preliminary processing of the TOC data collected from the three environments, the Ime4 package in R (V4.0.5) was used to analyze the correlation between TOC across different populations and environments. The mean, standard deviation, skewness, kurtosis, and coefficient of variation of the TOC were calculated, and a normal distribution curve was drawn. Broad-sense heritability was determined by using the method described by Knapp et al. [44].

(1) $$h^{2}=\frac{{\sigma g}^{2}}{{\sigma g}^{2}+{\sigma ge}^{2}/e+{\sigma \varepsilon }^{2}/re}\times 100\%$$

where ${\sigma g}^{2}$ refers to genetic variance, ${\sigma ge}^{2}$ refers to variance attributed to interactions between the genotype and environment, ${\sigma \varepsilon }^{2}$ refers to residuals, e refers to the environment, and r refers to replicates [44]. Broad-sense heritability ($h^{2}$) can help identify the variation in phenotypic traits. A higher $h^{2}$ indicates that the trait is primarily controlled by genetic factors and less affected by environmental factors.

### 4.4. DNA Extraction and Genotyping-by-Sequencing (GBS)

Genomic DNA was extracted from young maize leaves during the reproductive stage using the cetyl trimethyl ammonium bromide (CTAB) method [45,46]. The isolated DNA from each RIL was digested with restriction endonucleases PstI and MspI (New England BioLabs, Ipswich, MA, USA) and ligated with barcode adapters using T4 ligase (New England BioLabs). DNA libraries were constructed and sequenced following the GBS protocol.

All samples were purified using the QIAquick PCR purification kit (QIAGEN, Valencia, CA, USA). The polymerase chain reaction (PCR) was conducted using primers complementary to the two adapters. The PCR products were purified and quantified using the Qubit dsDNA HS Assay Kit (Life Technologies, Grand Island, NY, USA). After selecting 200–300 bp PCR products using the Egel system (Life Technologies), the concentration of each library was estimated using a Qubit 2.0 fluorometer and a Qubit dsDNA HS assay kit (Life Technologies). Template preparation and library sequencing were performed using the Ion PI HiQ Chef Kit (Thermo Fisher, Waltham, MA, USA). Sequencing was conducted on a P1v3 chip using the Ion Proton sequencer (Life Technologies, software version 5.10.1) [47]. The Ion Proton system produced sequencing reads of variable lengths. Following sequencing, raw reads were filtered to remove adaptor sequences and low-quality reads. Subsequently, clean reads were generated [48]. The maize B73 (RefGen_v4) genome was used as a reference for alignment during mapping, and the Sentieon software (v2021-12-01) was used for analysis (parameter “bwa mem-k 32-M-R”) [49]. SAMtools was used to convert the alignment results into SAM/BAM files, and SNP calling was performed using Genome Analysis Toolkit (GATK ) software (v4.2). SNPs were filtered based on a minimum allele frequency (MAF) threshold of ≥0.05, and SNPs with missing rates exceeding 10% were excluded. A total of 584,847 high-quality SNPs were generated and annotated using ANNOVAR software (v2013-05-09) [50].

### 4.5. Phylogenetic Tree, PCA, and Linkage Disequilibrium Analysis

Phylogenetic analysis was conducted using Tassel v5.0, using 584,847 high-quality SNPs to evaluate the genetic relationships among the 429 RILs in the multiparent population.

Principal component analysis (PCA) was performed using R v4.3.2, and the results were visualized using the scatterplot3d (v0.3.42) package.

Genome-wide SNPs were used to evaluate LD decay using PopLDdecay v3.42 software [51]. Default parameters were used to calculate r^2^ values, which measure the degree of linkage disequilibrium (LD) between markers. The r^2^ values range from 0 to 1, with values closer to 1 indicating a stronger LD between loci. The LD decay graph was generated using the Plot_OnePop.pl software (v2016-04-22) package.

### 4.6. Genome-Wide Association Analysis

GWAS for TOC was performed based on the mean TOC values of 429 RILs of the MPP in three different environments. BLUP values were also used during GWAS to identify SNPs significantly associated with TOC. GWAS was performed using the efficient mixed-model association (EMME) analysis implemented in the GEMMA (genome-wide efficient mixed-model association) package (v0.98.3) [52]. The mixed linear model analysis was performed using the following formula:

y = Xa + Sb + Km + e(2)

where y represents the phenotype, a and b are fixed effects representing labeled and unlabeled effects, respectively, and m represents unknown random effects. The incidence matrices a, b, and m are represented by X, S, and K, respectively, and e is the vector of the random residual effect. The population structure was corrected using the S-matrix, calculated from the first three principal components (PCs). The kinship (K) matrix was calculated using the simple matching coefficient matrix. The genetic relationships between individuals were modeled as random effects using the K matrix. Both population structure and kinship matrix were included as covariates during GWAS to reduce false positives.

Additionally, the lme4 package (version 1.1–30) in R [53] was used to calculate the BLUP values.

PLINK [54] was used to compute an independent marker using the parameter-independent pairwise 50 5 0.2. The significance threshold for identifying significant SNPs, −log10(p) > 4.5, was calculated using the formula −log10 (1/total SNP number) and the SNPs significantly associated with maize TOC were identified. SNPs meeting or exceeding the threshold were extracted using bedtools v1.7 [55]. Since linkage disequilibrium (LD) decayed at 20 kb, candidate genes associated with TOC were identified by screening regions 20 kb upstream and downstream of significantly associated SNPs, using available functional annotation information. Manhattan plots were generated to show the distribution of markers, and the Q-Q plot was used to evaluate the accuracy of the association analysis results.

### 4.7. Construction of Genetic Map and QTL Mapping

Genetic linkage maps for the five RIL subpopulations were constructed using polymorphic SNPs between the respective parents of the RIL subpopulations using JoinMap4.0 [56]. Linkage groups were established using LOD thresholds ≥ 2.5. QTLs for TOC were identified using the composite interval mapping (CIM) method in Windows QTL Cartographer v2.0 [57]. The LOD threshold was determined using 1000 random permutation tests at a significance level of *p* ≤ 0.05. QTLs with an LOD of ≥2.5 were considered significant. The percentage of phenotypic variation explained (PVE) by individual QTLs was calculated using the square of the partial correlation coefficient (R2). QTL names were assigned by starting with the letter ‘q’ to indicate the QTL, followed by the trait abbreviation, chromosome number, and marker position [58].

### 4.8. Identification and Functional Annotation of Candidate Genes

The loci consistently identified across different environments during GWAS were compared with the QTL mapping results to determine the SNPs that overlapped within the QTL interval. These overlapping SNPs were selected to screen for candidate genes. Candidate genes were identified within 20 kb upstream and downstream of significant SNPs. Gene predictions were based on the maize B73 v4 reference genome available in the MaizeGDB (https://www.maizegdb.org/, accessed on 15 November 2023). Furthermore, expression data of candidate genes regulating TOC at different time points or locations were extracted from the MaizeGDB database and compared. Functional annotation of the candidate genes was performed using the InterPro database.

### 4.9. Haplotype Analysis

Haplotype analysis of SNPs associated with candidate genes regulating TOC identified across the three environments was performed using the Haploview v4.2 software. First, a haplotype map was constructed using high-density genome-wide SNPs. Next, haplotypes containing significantly associated SNPs were identified based on the location of the significant loci associated with TOC and LD analysis. Finally, genes within the haplotypes were annotated to identify functionally related gene loci.

## 5. Conclusions

In this study, five RIL subpopulations were constructed by crossing the temperate inbred line Ye107 with subtropical inbred lines CML312 and CML384 and tropical inbred lines CML395, YML46, and YML32. A total of 18 QTLs and 60 SNPs associated with maize TOC were identified. Subsequently, two TOC-related candidate genes, *Zm00001d029550* and *Zm00001d029551*, were identified by screening 20 Kb upstream and downstream regions of these SNPs. The SNP-75791466 was located 5648 bp downstream of *Zm00001d029550* and 11,951 bp downstream of *Zm00001d029551*. These two candidate genes are believed to be involved in the genetic mechanisms underlying TOC in maize. By introducing temperate and tropical germplasms, a novel QTL, *qTOC1-2*, was identified on chromosome 1 with an LOD of 3.82 and a PVE of 1.4%. The results indicated the presence of novel genes related to maize oil content in the tropical germplasm. In this study, colocalized loci were identified by linkage analysis and GWAS, highlighting the reliability of the two candidate genes. This provides a foundation for further research to validate these genes and the application of genomic selection in breeding high-oil maize varieties. In summary, the findings of this study enhance our understanding of the regulatory mechanisms underlying TOC in maize, and the SNP and candidate genes identified are expected to help breeders in developing high-oil maize varieties.

## Figures and Tables

**Table 1:** Statistical analysis of TOC in five RIL subpopulations conducted in three environments.

Population	Environment	Mean	Standard Deviation	Skewness	Kurtosis	Coefficient of Variation (%)	Heritability (*h*^2^) (%)	Correlation Coefficient
								(r)
pop1	22YS	5.273	0.662	−0.158	−0.481	12.60%	88.1	22YS/23JH = 0.77 **
	23JH	5.189	0.623	0.375	−0.226	12%		23JH/21YS = 0.73 **
	21YS	5.199	0.711	0.070	−0.080	13.70%		21YS/22YS = 0.68 **
	total	5.220	0.665	0.079	−0.286	12.74%		
pop2	22YS	5.164	0.590	0.146	−0.010	11.40%	93.1	22YS/23JH = 0.89 **
	23JH	5.050	0.550	0.314	−0.334	10.90%		23JH/21YS = 0.91 **
	21YS	4.910	0.610	0.203	−0.217	12.40%		21YS/22YS = 0.72 **
	total	5.041	0.591	0.185	−0.192	11.72%		
pop3	22YS	5.501	0.809	−0.243	−0.071	14.70%	83.9	22YS/23JH = 0.87 **
	23JH	5.320	0.623	0.052	−0.441	11.70%		23JH/21YS = 0.79 **
	21YS	5.120	0.620	0.019	−0.387	12.10%		21YS/22YS = 0.47 **
	total	5.313	0.704	0.041	−0.160	13.25%		
pop4	22YS	4.884	0.432	0.533	−0.045	8.80%	79.4	22YS/23JH = 0.78 **
	23JH	4.888	0.371	0.163	−0.925	7.60%		23JH/21YS = 0.76 **
	21YS	4.912	0.439	0.353	−0.087	8.90%		21YS/22YS = 0.40 **
	total	4.895	0.413	0.378	−0.233	8.44%		
pop5	22YS	5.276	0.592	0.241	−0.402	11.20%	91.6	22YS/23JH = 0.91 **
	23JH	5.214	0.542	0.242	−0.644	10.40%		23JH/21YS = 0.88 **
	21YS	5.167	0.504	0.222	−0.274	9.80%		21YS/22YS = 0.70 **
	total	5.216	0.547	0.263	−0.412	10.49%		

Note: 22YS refers to the trials conducted in Yanshan in 2022, 23JH denotes the trial conducted in Jinghong in 2023, and 21YS represents the trials conducted in Yanshan in 2021. ** indicates *p* < 0.01.

**Table 2:** Details of SNPs significantly associated with TOC identified in GWAS (B73 (RefGen_v4)).

Env.	Chr.	SNP	ref	alt	−log(P)	Additive Effect	Dominance Effect	PVE
22YS	1	75,791,466	G	T	5.23	−0.26	0.35	0.035
22YS	3	2,108,126	G	A	4.80	0.20	0.20	0.075
22YS	3	7,453,745	G	A	5.46	0.30	0.04	0.053
22YS	3	8,646,933	C	A	6.15	0.33	−0.11	0.046
22YS	3	9,226,566	G	T	4.54	−0.28	−0.21	0.036
22YS	3	9,371,935	C	T	5.14	−0.29	−0.20	0.038
22YS	3	230,340,051	C	T	4.68	−0.24	0.14	0.062
22YS	3	230,499,437	G	A	4.57	0.25	0.04	0.028
22YS	4	52,876,804	A	G	4.77	−0.19	−0.48	0.035
22YS	4	203,717,068	A	G	6.45	−0.41	−0.01	0.073
22YS	5	216,419,106	A	T	5.30	−0.26	−0.05	0.049
22YS	6	19,088,018	C	T	4.63	NaN	NaN	0.029
22YS	7	140,826,856	C	T	4.94	0.31	0.06	0.042
22YS	8	1,952,449	C	T	5.22	0.15	−0.13	0.038
22YS	8	172,972,407	T	C	4.99	−0.27	0.13	0.055
22YS, BLUP	8	173,247,098	A	T	6.86	−0.37	−0.18	0.070
22YS, 23JH, BLUP	8	174,055,891	T	C	6.32	−0.27	−0.48	0.098
22YS	8	177,414,430	C	T	4.71	−0.22	−0.01	0.077
22YS	9	13,835,261	C	T	4.72	−0.25	0.00	0.092
22YS, 23JH, BLUP	9	14,820,336	C	A	5.25	0.24	−0.14	0.055
22YS, 23JH, BLUP	9	92,493,718	T	C	5.24	−0.57	−0.16	0.053
22YS	10	115,482,753	G	A	4.67	0.23	0.27	0.110
22YS	10	138,012,512	T	G	5.23	0.35	−0.61	0.117
23JH	1	89,810,991	G	T	4.61	−0.36	−0.11	0.053
23JH	2	172,364,269	T	C	5.02	NaN	NaN	0.083
23JH, BLUP	4	80,064,051	C	A	4.84	0.26	0.12	0.034
23JH, BLUP	9	110,672,521	T	C	4.83	−0.25	0.21	0.071
23JH, 21YS	10	17,500,491	C	G	4.85	NaN	NaN	0.057
21YS	2	5,102,776	G	T	5.08	−0.27	−0.58	0.064
21YS	2	62,659,851	G	A	5.21	NaN	NaN	0.088
21YS	4	131,018,543	C	A	5.40	NaN	NaN	0.072
21YS	4	132,312,280	C	T	4.76	NaN	NaN	0.055
21YS	5	221,373,665	G	A	6.13	0.22	−0.01	0.059
21YS	5	222,162,464	T	C	4.70	0.17	−0.01	0.044
21YS	7	21,816,794	C	G	5.00	0.24	−0.13	0.073
21YS	7	165,218,537	A	G	5.96	0.26	−0.17	0.084
21YS	9	40,627,693	G	A	5.45	NaN	NaN	0.075
21YS	9	54,914,157	T	C	4.64	NaN	NaN	0.057
21YS	9	108,901,209	A	G	4.89	0.20	0.34	0.053
21YS, BLUP	9	108,933,426	A	G	7.44	0.29	−0.09	0.108
21YS	9	109,017,561	A	G	5.50	0.21	−0.15	0.075
21YS	9	109,122,650	G	A	4.58	−0.20	0.14	0.080
21YS	9	109,283,271	G	A	4.94	−0.16	−0.34	0.086
21YS	9	109,407,646	C	T	5.07	0.22	0.16	0.063
21YS	9	109,451,171	G	C	4.94	−0.20	0.09	0.057
21YS	9	110,611,574	G	T	5.70	0.29	−0.32	0.132
21YS	9	110,672,521	T	C	5.71	−0.25	−0.19	0.110
21YS	10	17,500,491	C	G	4.60	NaN	NaN	0.074
BLUP	7	142,297,954	A	G	4.83	0.19	−0.09	0.076
BLUP	9	108,933,426	A	G	4.51	0.21	−0.03	0.081
BLUP	9	152,304,134	T	A	4.94	0.17	−0.11	0.056

Note: Env: environment; Chr: chromosome.

**Table 3:** Positions and effects of TOC-linked QTLs detected in four RIL subpopulations.

Mapping Population	QTL	Chromosome	Position (cM)	Mapping Interval (bp)	LOD	Additive Effect	R^2^
pop1	qTOC2-1	2	115	29,801,036–37,206,712	2.90	0.163	0.090
	qTOC2-2	2	106.98	14,177,571–47,384,437	4.63	0.130	0.060
	qTOC2-3	2	117.03	14,177,571–37,206,712	3.75	0.130	0.050
	qTOC3-1	3	33.25	193,353,260–227,027,669	3.82	−0.098	0.030
	qTOC9-1	9	75.92	110,367,967–125,469,868	3.39	−0.106	0.030
pop3	qTOC1-1	1	27.11	273,307,800–273,997,185	3.81	0.400	0.131
	qTOC1-2	1	198.72	61,742,795–76,153,784	3.82	0.250	0.014
	qTOC4-1	4	232.73	161,621,304–164,227,248	5.65	0.650	0.199
	qTOC4-2	4	599.88	172,418,126–175,502,163	3.46	0.320	0.143
	qTOC7-1	7	199.1	50,416,681–50,735,457	4.33	−0.560	0.151
pop4	qTOC5-1	5	34.22	79,346,283–81,012,543	3.31	0.038	0.005
	qTOC7-1	7	29.17	132,794,775–153,544,218	3.01	−0.146	0.110
	qTOC8-1	8	89.73	10,663,910–30,775,820	4.61	−0.166	0.124
pop5	qTOC2-1	2	1.57	222,088,040–232,416,169	3.75	−0.290	0.144
	qTOC2-2	2	15.67	159,679,997–163,407,700	5.73	0.360	0.231
	qTOC3-1	3	33.53	114,248,818–166,417,378	4.27	−0.240	0.177
	qTOC5-1	5	22.99	135,409,128–153,772,976	3.92	0.280	0.146
	qTOC5-2	5	55.99	31,369,431–38,866,833	2.80	−0.230	0.103

**Table 4:** Candidate genes identified through combined GWAS and QTL mapping analyses.

SNP/QTL	Chromosome	Position	Mapping Interval (bp)	Candidate Gene	Candidate Gene Range (bp)	Gene Annotation
SNP-75791466	1	75,791,466 bp	[75,771,466–75,811,466]	*Zm00001d029550*	75,797,114–75,806,603	Diacylglycerol Kinase 1
qTOC1-2	1	198.72 cM	61,742,795–76,153,784			
SNP-75791466	1	75,791,466 bp	[75,771,466–75,811,466]	*Zm00001d029551*	75,803,417–75,804,837	NaN
qTOC1-2	1	198.72 cM	61,742,795–76,153,784			

**Table 5:** Key haplotypes associated with TOC in maize.

Gene ID	SNP Position	Haplotype	Hap_Sample_Num ^1^
*Zm00001d029550*	Chr1: 75,791,466 bp	GTTCTACG(Hap1)	192
		ATCTGGTA(Hap2)	74
		ACCTGGTA(Hap3)	32
*Zm00001d029551*	Chr1: 75,791,466 bp	GTTCT(Hap1)	129
		ATCTG(Hap2)	126
		ACCTG(Hap3)	40

Note: ^1^ hap_sample_num refers to the total number of identical haplotypes.

**Table 6:** Research progress of TOC-related QTLs in maize.

Materials	Population Type	Trait	QTL	Marker/Physical Interval	LOD	PVE (%)	Reference
B73, By804	RIL	KO	qKO1-1	umc1598–umc1884	-	14.3	[25]
Ku13, Sc55	RIL	OIL	qOLE1-1	66.4–71.0 Mb	4.84	11.06	[8]
B73, By804	RIL	OIL	OIL1-1	umc2217–bnlg2086	-	-	[11]
B73, By804	RIL	EER	qEEWR1-1	73,374,836–73,376,998 bp	-	-	[26]

Note: KO represents the kernel oil content, OIL represents the oleic, and EER represents the embryo-to-endosperm ratio.

**Table 7:** Parental lines used to develop the multiparent population.

Parent	Pedigree	Heterotic Group	Ecological Type	Total Oil Content (%)
Ye107	Derived from US hybrid DeKalb XL80	Reid	Temperate	3.51
CML312	S89500-F2-2-2-1-1-B*5-2-1-6-1(DH)	nonReid	Subtropical	6.8
CML384	P502c1#-771-2-2-1-3-B-1-1-3-1(DH)	Reid	Subtropical	7.03
CML395	90323B-1-B-1-B*4-1-1-2-1(DH)	nonReid	Tropical	7.1
YML46	SW1-1-1-2-1-2-1	Suwan	Tropical	5.9
YML32	Suwan 1(S)C9-S8-346-2 (Kei 8902)-3-4-4-6	Suwan	Tropical	7.3

* represents five generations of mixed threshing of all fruit ears. # represents mixed pollination of all fruit clusters.

## Data Availability

The datasets for this study can be found in the China National Center for Bioinformation with the BioProject ID PRJCA030593.
